# Collagen fingerprinting and sequence analysis provides a molecular phylogeny of extinct Australian megafauna

**DOI:** 10.1098/rspb.2025.0856

**Published:** 2025-11-12

**Authors:** Michael Buckley, Kieren J. Mitchell, Lee J. Arnold, Elizabeth H. Reed, Rolan Eberhard

**Affiliations:** ^1^ Earth and Environmental Sciences, University of Manchester, Manchester, UK; ^2^ Bioeconomy Science Institute, Lincoln, New Zealand; ^3^ School of Biological Sciences, University of Adelaide, Adelaide, South Australia, Australia; ^4^ School of Physics, Chemistry and Earth Sciences, Institute for Photonics and Advanced Sensing, University of Adelaide, Adelaide, South Australia, Australia; ^5^ School of Biological Sciences, University of Adelaide, Adelaide, South Australia, Australia; ^6^ Tasmanian Museum and Art Gallery, Hobart, Tasmania, Australia

**Keywords:** ancient collagen, ZooMS, Tasmania, Sahul, Australian megafauna, extinction debates

## Abstract

During the Late Pleistocene, Sahul—the former land mass of Australia, Tasmania and New Guinea—faced one of the greatest waves of megafaunal extinctions on the planet, for reasons that remain highly debated. Yet how some of these extinct species relate to each other also remains unclear, with poor DNA preservation causing challenges for reconstructing phylogenies of extinct taxa using biomolecular data. Here, we use ZooMS collagen peptide mass fingerprinting to screen 51 marsupial bones from Tasmania, ranging in age from late Holocene to over 100 000 years old, to locate specimens of extinct megafauna with the best potential for peptide sequence analysis. We then carried out phylogenetic analyses of collagen peptide sequences, providing the first biomolecular evidence for the relationships of the extinct marsupial genera *Zygomaturus*, *Palorchestes* and *Thylacoleo*. Most notably, our collagen data raise the possibility that the koala (*Phascolarctos cinereus*) may be the closest living relative of *Thylacoleo carnifex*, the so-called ‘marsupial lion’. Furthermore, by yielding biomolecular data from specimens that far pre-date human arrival, our study demonstrates that ZooMS can be an important tool for establishing higher-resolution extinction chronologies for extinct megafauna from Sahul, which may help to more conclusively establish the cause of their extinction.

## 1. Introduction

### (a) Extinctions of the Late Pleistocene Sahul megafauna

In our relatively recent past, during the Late Pleistocene (approx. 130 ka to approx. 12 ka), a large proportion of megafaunal biodiversity was lost worldwide [1]. No land mass was more affected than Australia, where nearly 90% of terrestrial species over 44 kg in body weight were lost by ~46 ka [2], roughly coinciding with the arrival of humans to the continent (as early as 65 ka [3]). Extinction of the Australian megafauna strongly affected the continent’s biodiversity and represented a major loss in evolutionary history, the causes of which remain poorly understood [4–6].

Among the extinct megafaunal taxa were many species of giant kangaroo, including those belonging to *Simosthenurus* and *Protemnodon* [7,8]. These extinctions also wiped out other more enigmatic, non-macropodid taxa such as *Palorchestes azael*, a herbivorous marsupial with a body weight of approximately 1000 kg, fixed elbow joints and unusual rostral morphology [9,10], interpreted as a tapir-like snout or prehensile lip with a long tongue [11]. Another species lost during the Late Pleistocene was *Thylacoleo carnifex*, a hyper-carnivore with body weight above 100 kg [12], with large blade-like premolars and exceptionally high bite force [13]. *Thylacoleo* was especially notable, as its ancestors appear to have evolved carnivory independently from members of Dasyuromorphia, the order that includes smaller marsupial carnivores like the Tasmanian devil (*Sarcophilus harrisii*) and thylacine (*Thylacinus cynocephalus*). These Late Pleistocene extinctions encompassed all of the largest browsers, grazers, predators, anteaters and scavengers in Sahul—the formerly contiguous land mass of mainland Australia, Tasmania and New Guinea—with major ecological consequences including profound changes to vegetation structure [14,15]. Yet the exact timing and cause of these extinctions—whether primarily due to climate change, human activities, or a combination of the two—remain highly debated [16,17].

Based on youngest dated fossils, extinction of the Sahul megafauna was a staggered process culminating in a peak of extinctions between 50 ka and 40 kaa on mainland Australia and somewhat later in Tasmania [5,6]. However, for some megafaunal taxa, this conclusion is based on small numbers of reliably identified and dated specimens [18]. Recent years have seen great improvements in biomolecular methods of species identification that could be key to tackling this question. This has involved advances in genetic research used for faunal identifications at Australian sites with exceptional preservation conditions [8] as well as complementary advances in proteomic research, particularly the application of ZooMS [19]. These methods have been applied to megafaunal remains globally [20–22], including extinct kangaroos [23] and other marsupials [24].

One of the strengths of biomolecular sequence information retrieved from ancient specimens is the improved phylogenetic resolution it provides, circumventing issues with using morphology alone to establish taxonomic and evolutionary relationships (see [25,26]). This is particularly relevant to many extinct Australian megafauna that lack any biomolecular data, including the morphologically unusual *Palorchestes azael* (Diprotodontia: Vombatiformes: Palorchestidae), the giant herbivore *Zygomaturus trilobus* (Diprotodontia: Vombatiformes: Diprotodontidae) and the apex predator *Thylacoleo carnifex* (Diprotodontia: Vombatiformes: Thylacoleonidae). The phylogenetic relationships of these extinct vombatiform families—and their extant relatives—have historically been difficult to reconstruct for several reasons. First, a 30 million-year gap in the Australian marsupial fossil record between the early Eocene and late Oligocene obscures the differentiation of many Australian marsupial families [27]. Furthermore, late Oligocene representatives of these families often already had highly derived (autapomorphic) anatomical features, making them difficult to group based on morphological similarity [28,29]. Finally, poor DNA preservation has meant that extinct Pleistocene marsupials have been omitted from molecular phylogenetic studies [30]; the only exceptions being two extinct species of macropodids, *Simosthenurus occidentalis* and *Protemnodon anak* [8,31].

### (b) Ancient biomolecules for taxonomic identification and molecular phylogenies

The advent of reliable methods for biomolecular identification of ancient bone has created scope for increased confidence in evaluating faunal assemblages in Late Pleistocene palaeontological and archaeological contexts [22]. Application of these methods has the potential to overcome the perennial problem that a proportion of material within most ancient bone assemblages is fragmented, weathered or otherwise modified to the extent that critical morphological diagnostic features have been destroyed. Relying on only physical characteristics to analyse biological samples is likely to limit the amount and quality of information that can be gained from them (see [26]), with numerous studies emphasizing the importance of at least including molecular data for improving phylogenetic trees [25].

Within certain limitations, recovery of ancient DNA (aDNA) has emerged as an effective tool for identifying otherwise ambiguous samples [32], but has well-known preservation limitations relating to time and temperature, most often not reaching as far into the past as preferred for studies into early human–animal interactions or palaeoecological studies prior to human arrival. An alternative method—collagen fingerprinting—has been refined for application to the identification of ancient bones. The collagen fingerprinting technique, also referred to as zooarchaeology by mass spectrometry (ZooMS), is diagnostic at genus and species levels for many groups [33]. The proteins used in generating collagen fingerprints are less prone to degradation than DNA molecules, implying the possibility of their preservation in samples that are not suitable for aDNA. Due to the rapid and robust nature of the method, collagen fingerprinting can also be used as a screening tool for biomolecular preservation, such as radiocarbon dating [34]. Ultimately, it is differences in the amino acid sequence that causes different collagen Peptide Mass Fingerprints (PMFs), mirroring evolutionary relationships between the species to a somewhat limited extent [35]. More importantly, both as a tool for screening sample quality and improving confidence in peptide sequence assignment to a given taxon, collagen fingerprinting has widespread application to resolving phylogenetic relationships [35–37], that has not yet been applied to yield a molecular phylogeny of extinct Australian megafauna.

Due to the early extinctions of megafauna in Sahul and the poor biomolecular preservation conditions of the Australian marsupial fossil record, driven by a particularly warm climate across much of the continent, aDNA has been of limited use for establishing richer extinction chronologies. Therefore, the primary aims of this study were to use collagen PMF to screen a broad suite of iconic extinct Australian megafauna from sites that span over 100 000 years and then obtain peptide sequence data for reconstructing a molecular phylogeny that includes *Zygomaturus*, *Palorchestes* and *Thylacoleo*, as well as the extinct macropodids *Protemnodon* and *Simosthenurus*.

## 2. Material and methods

### (a) Sample sites

Tasmania (42°S, 146°E) lies at the southeastern extremity of the former Sahul and present-day Australia. Separated from mainland Australia by the 200 km wide Bass Strait, Tasmania has a cool temperate maritime climate strongly influenced by dominantly westerly ‘Roaring Forties’ winds off the Southern Ocean. Tasmania’s lower temperature (cave temperatures typically 8–9°C) and the presence there of karst caves with well-preserved marsupial fossils underpinned our decision to sample Tasmanian material. The sampled specimens were sourced chiefly from pitfall caves, but they also include a former carnivore den and a swamp (electronic supplementary material, table S1).

All sites are, or were, located in tall wet forest (electronic supplementary material, figure S1), prior to conversion of some to agricultural land or plantation forests in the twentieth century. A mix of extinct Late Pleistocene species and extant Holocene species was sampled, to build a more comprehensive library of marsupial collagen fingerprints.

#### (i) Scotchtown Cave

Scotchtown Cave lies at 30 m above sea level (ASL) in northwest Tasmania. The site came to attention in 1942 when a limestone quarry intersected a red earthy cave fill containing abundant bones. A selection of bones was collected by staff from the Queen Victoria Museum, Launceston before the site was quarried out of existence (e.g. electronic supplementary material, figure S2). The fossil assemblage is noteworthy for the diversity of species present and the broken-up condition of the bones, suggesting the cave was formerly an animal den [38]. Samples (SC/2022/1−13) from Scotchtown Cave were preserved within a body of sediment for which there is no stratigraphic description and minimal information generally. Sediment grains taken from a ‘mixed soil-bone’ sample held by the Queen Victoria Museum and Art Gallery returned an optically stimulated luminescence (OSL) age of 56 ± 4 ka [39]. Whether this result is a reliable proxy for the age of the bones is uncertain because the OSL analysis relied on ex situ material, precluding application of standard field dosimetry measurements. Three samples of emu bone from Scotchtown Cave have returned ^14^C ages [40] that calibrate in the range 38.2−42.6 ka cal BP (OxCal v4.4 SHCal 20 calibration curve). These suggest that elements of the Scotchtown Cave fossil assemblage accumulated towards the middle of MIS3, in broad alignment with the assumed timing of megafauna extinctions in Tasmania at ~40 ka [39,41].

#### (ii) Devils Earhole

Devils Earhole is a large cavern at 600 m ASL on the flanks of the Great Western Tiers of central northern Tasmania. The age of the bone in our sample is not known, but likely of similar age to other bones from this cave, which have returned ages of >2 ka to modern [42].

#### (iii) Femur Fest Cave, Emu Cave and Cave JF191

These small caves are found at 380 m ASL in the Florentine Valley. Femur Fest Cave and Emu Cave contain megafauna bones buried in silty sediment, the age of which is constrained at Femur Fest Cave by single-grain OSL dating, as reported below. Importantly, OSL dating of the host deposits constrains the age of sample JF643/5 (electronic supplementary material, figure S3). Cave JF191 is a pitfall cave for which no radiometric ages have yet been reported. The bone in our sample was found resting on the base of the cave in relatively un-weathered condition, suggesting a Holocene age. Samples EC/2015/1, EU/2016/24, EU/2016/301, EU/2016/3025−6 from Emu Cave also include extinct megafauna and are therefore unlikely to be much younger than ~40 ka.

#### (iv) Predator Pot

Predator Pot (560 m ASL) is a pitfall cave on the flanks of the Mt Field Range. It contains bones of extinct and extant marsupials, the remains of which are scattered on the base of the cave and buried at shallow depth in clayey sediment. No ages have yet been reported but the site is unlikely to be much younger than ~40 ka based on extinction timing for megafauna in Tasmania [41]. Likewise, samples JF676/2019/1H, JF676/2019/3, JF676/2019/8 and CC/2018/5 from Predator Pot also include extinct megafauna and are therefore unlikely to be much younger than ~40 ka.

#### (v) Mowbray Swamp

Mowbray Swamp occupies a low-lying coastal plain where clusters of karstic spring mounds have created waterlogged condition, prior to conversion of the swamp to agricultural land in the early twentieth century. The drainage works exposed articulated skeletons of *Z. trilobus*, preserved in sapric peat up to 2 m deep. Fossil bones from Mowbray Swamp are darkly stained compared to the aforementioned cave bones (electronic supplementary material, figure S4), suggesting that contamination by organic compounds within the peat has conditioned their preservation. We recorded very low soil pH conditions (pH 3−5) during site visits. Samples (MS/2022/1−5) from Mowbray Swamp are not tightly constrained in their age. Attempts to directly date megafauna bones from the swamp [41] have returned ages close to or exceeding the practical limits of the radiocarbon method (i.e. ~50 ka). Peat and wood from the swamp have likewise typically returned infinite radiocarbon dates [43,44]. The peat in which the bones were found is underlain by Last Interglacial marine sand [38], implying that the bones must be younger than ~116 ka.

### (b) Faunal remains

For ZooMS analyses, we tested 51 fossil bones from six Tasmanian caves and one swamp deposit (electronic supplementary material, table S1); Scotchtown Cave (*n* = 33), Emu Cave (*n* = 5), Predator Pot (*n* = 2), Devils Earhole (*n* = 1), Femur Fest Cave (*n* = 4), Cave JF191 (*n* = 1) and Mowbray Swamp (*n* = 5). Morphological identifications were based on museum registrations or comparison with confidently identified reference specimens. Doubtful and unidentified specimens were given ‘indeterminate’ status (electronic supplementary material, table S2). Collectively these encompass confidently identified specimens including *Macropus giganteus*, *Protemnodon sp.*, *Palorchestes azael*, *Z. trilobus*, *S. occidentalis*, *Thylogale billiardierii*, *Vombatus ursinus* and *Notamacropus rufogriseus*. We also included five indeterminate specimens, 11 assigned to an unresolved taxon (i.e. ‘large macropod’), and two speculative attributions to *Thylogale billardierii* to test the species identification capabilities of ZooMS collagen PMF for the marsupial megafaunal remains. The samples were obtained by drilling except for a few weathered specimens where flaking chips of bones were taken.

### (c) Collagen fingerprinting and sequencing

Collagen extraction was attempted from ~25 to 50 mg of each specimen using 0.6 M hydrochloric acid for approximately 18 hours and the acid-soluble collagen transferred into 50 mM ammonium bicarbonate using 10 kDa ultrafilters. Samples were then digested with 0.4 µg sequencing grade trypsin (Promega, UK) overnight at 37°C. All of the sample digests were initially spotted directly onto the MALDI plate upon 1/20 dilution in 0.1% TFA, while the 10 samples selected for LC-MS/MS were then fractionated into 10 and 50% acetonitrile (ACN; in 0.1% trifluoroacetic acid; TFA) using C18 ZipTips (OMIX, UK), evaporated, resuspended in 0.1% TFA and spotted onto the stainless-steel target plate for fingerprint analysis using a Brüker Rapiflex Matrix Assisted Laser Desorption Ionization Time of Flight (MALDI-ToF) mass spectrometer. Half of each aliquot (10 and50% ACN fractions, of the selected 10 samples) was then combined, and after being evaporated to completion and resuspended with 20 µl 5% ACN/0.1% formic acid (FA), 2 µl was subjected to in-depth sequencing by LC-Orbitrap Elite mass spectrometric analysis. Sequencing was carried out using an UltiMate 3000 Rapid Separation LC (RSLC, Dionex Corporation, Sunnyvale, CA, USA) coupled to an Orbitrap Elite (Thermo Fisher Scientific, Waltham, MA, USA) mass spectrometer (120 k resolution, full scan, positive mode, normal mass range 350−1500) following analytical methods described by Wadsworth and Buckley [45]. Primarily, sequences were recovered via several rounds of error tolerant searches against a local database, both automated (using Mascot, UK) and manual [46] that included the concatenated COL1A1 and COL1A2 sequences [47] for eight marsupial sequences available from a protein BLAST search of wombat collagen (electronic supplementary material, table S3); for further details see electronic supplementary material, text S1.

### (d) Phylogenetic analyses

These sequences (electronic supplementary material, text S2) were then ordered by position and manually aligned in BioEdit Sequence Alignment Editor v7.1.3.0 with an ‘X’ representing unknown/unmatched amino acid residues following Buckley [35]. We then created a maximum likelihood phylogeny from our complete amino acid alignment using IQ-TREE v3.0.1 [48]. The optimal amino acid substitution model (mtMAM+F+I+R2) was determined using ModelFinder [49], and branch support was assessed using 1000 ultrafast bootstrap replicates [50]. We positioned the root of the tree between Monotremata (i.e. *Tachyglossus* and *Ornithorhynchus*) and the remaining sequences. We also calculated likelihood scores for 12 alternative tree topologies in which the phylogenetic positions for the extinct vombatiform taxa varied and compared these alternative topologies to the tree with the highest likelihood using the Approximately Unbiased (AU) test [51], as implemented in IQ-TREE (electronic supplementary material, table S4).

We co-estimated the phylogeny and divergence times under a Bayesian framework using BEAST2 v2.7.7 [52] for sequences belonging to Diprotodontia (the smallest clade comprising all our ancient sequences). We used OBAMA v1.1.1 [53] to average over a set of best-fitting amino acid substitution models (restricted to combinations of MtArt, MtMam, MtREV with or without estimated frequences, gamma and invariant sites). Our analysis included a relaxed log-normal clock model and a birth-death tree prior. To calibrate our phylogeny, we placed uniform age distributions (23.03–54.6 Ma) on the root of the tree and the origin of the branches leading to Macropodidae, Vombatidae, Diprotodontidae, Palorchestidae, Phascolarctidae and Thylacoleonidae. The 54.6 Ma maximum bound for the age of all calibrated nodes was based on the maximum age estimate for the Tingamarra Local Fauna, which includes several plesiomorphic marsupials but no identifiable crown diprotodontians [54]. The 23.03 Ma minimum bound for the age of all calibrated nodes was based on the presence of stem members of Macropodidae, Vombatidae, Diprotodontidae, Palorchestidae, Phascolarctidae and Thylacoleonidae in various Late Oligocene fossil assemblages [27]. We ran three independent Markov chain Monte Carlo instances, each 10 000 000 steps in length, sampling parameter values every 1000 steps. Convergence of parameters and effective sample sizes were assessed using Tracer v1.7.2. We combined all samples after discarding the first 10% from each chain as burn-in and created a maximum clade credibility tree. Combined effective sample sizes for all parameters were >500.

### (e) OSL dating

Five optical dating samples were collected from cleaned sedimentary exposures at Femur Fest Cave to provide an estimate of when the fossil-bearing infill deposits were last exposed to light prior to burial. Samples were extracted either as intact blocks of sediment (FF16-4b) or using metal tubes (FF16-1, FF16-2, FF16-3, FF16-5), and additional bulk sediment was collected from within a 1 cm radius of each sample position for water content and dosimetry evaluations. Four samples were collected from the 40−50 cm-thick bone breccia deposit (Unit 2a) that has yielded fossil dentary samples JF643/4 and JF643/5. Sample FF16-4b was taken from the uppermost 5 cm of this dark brown silty-clay bone breccia unit (Unit 2a), while samples FF16−2 and FF16−3 were taken from the upper and middle layers of the same bone breccia unit, approximately 19 cm and 25 cm below the contact with a thin (10–15 cm) capping orange clay unit (Unit 1). Sample FF16-5 was collected from the lowermost/basal layers of the main bone breccia unit (Unit 2a), providing an initial age for the fossil deposit. Finally, sample FF16-1 was taken 5 cm below the bone breccia unit from an underlying brown clay unit that contains fewer fossil remains (Unit 2b). The optical dating study focused on single-grain quartz OSL analyses (electronic supplementary material, figure S5) in order to gain further insights into potential methodological complications that can affect OSL dating reliability in cave settings; particularly the presence of insufficiently bleached grain populations (e.g. [55]) and contaminant grains associated with syn- or post-depositional mixing (e.g. [56]). Purified coarse-grain quartz extracts were processed under safe light conditions (630 nm LEDs < 0.15 μW cm^−2^ power density at sample position) using standard preparation procedures that included a 48% hydrofluoric acid etch (40 min) to remove the alpha-irradiated outer layers of the quartz extracts. Single-grain OSL measurements were made using the experimental apparatus, single-aliquot regenerative-dose (SAR) procedures which are further detailed in the electronic supplementary material, text S3. Between 700 and 900, single-grain equivalent dose (D_e_) measurements were made for each sample using the SAR procedure shown in electronic supplementary material, table S5, which yielded suitable dose-recovery test results for sample FF16.5 (electronic supplementary material, figures S6 and S7, tables S6 and S7).

Environmental dose rates were estimated using a combination of *in situ* field gamma spectrometry and low-level beta counting, taking into account cosmic ray contributions [57], an assumed minor internal alpha dose rate [58], beta-dose attenuation [59] and long-term sediment water contents [60,61]. Gamma dose rates were calculated from *in situ* measurements made at each sample position with a NaI:Tl detector (using the ‘energy windows’ approach [62]), while beta-dose rates were calculated on dried and powdered sediment collected from within a 1 cm radius of each sample position using a Risø GM-25-5 low-level beta counter [63].

## 3. Results

### (a) Chronological context

The four OSL ages obtained for the main fossil-bearing deposit (Unit 2a) at Femur Fest Cave are statistically indistinguishable from each other and indicate that the host sediments accumulated 89.3 ± 6.1 to 99.2 ± 6.3 ka, most likely during the Marine Isotope Stage 5 interglacial complex (130–71 ka [64]) (electronic supplementary material, table S1). An additional OSL age obtained from the immediately underlying (Unit 2b) deposits at Femur Fest Cave provides a maximum bracketing age of 101.7 ± 5.5 ka for the main fossil deposit (electronic supplementary material, table S1), confirming that the broader infill sequence accumulated relatively rapidly during MIS 5d-a. These data establish the upper and lower age limits of samples JF643/4 and JF/643/5. The ages of other samples in this study can be inferred with variable precision from field observations and published data (see §2).

### (b) Collagen preservation and species identification inferred through peptide mass fingerprints

**Figure 1.**
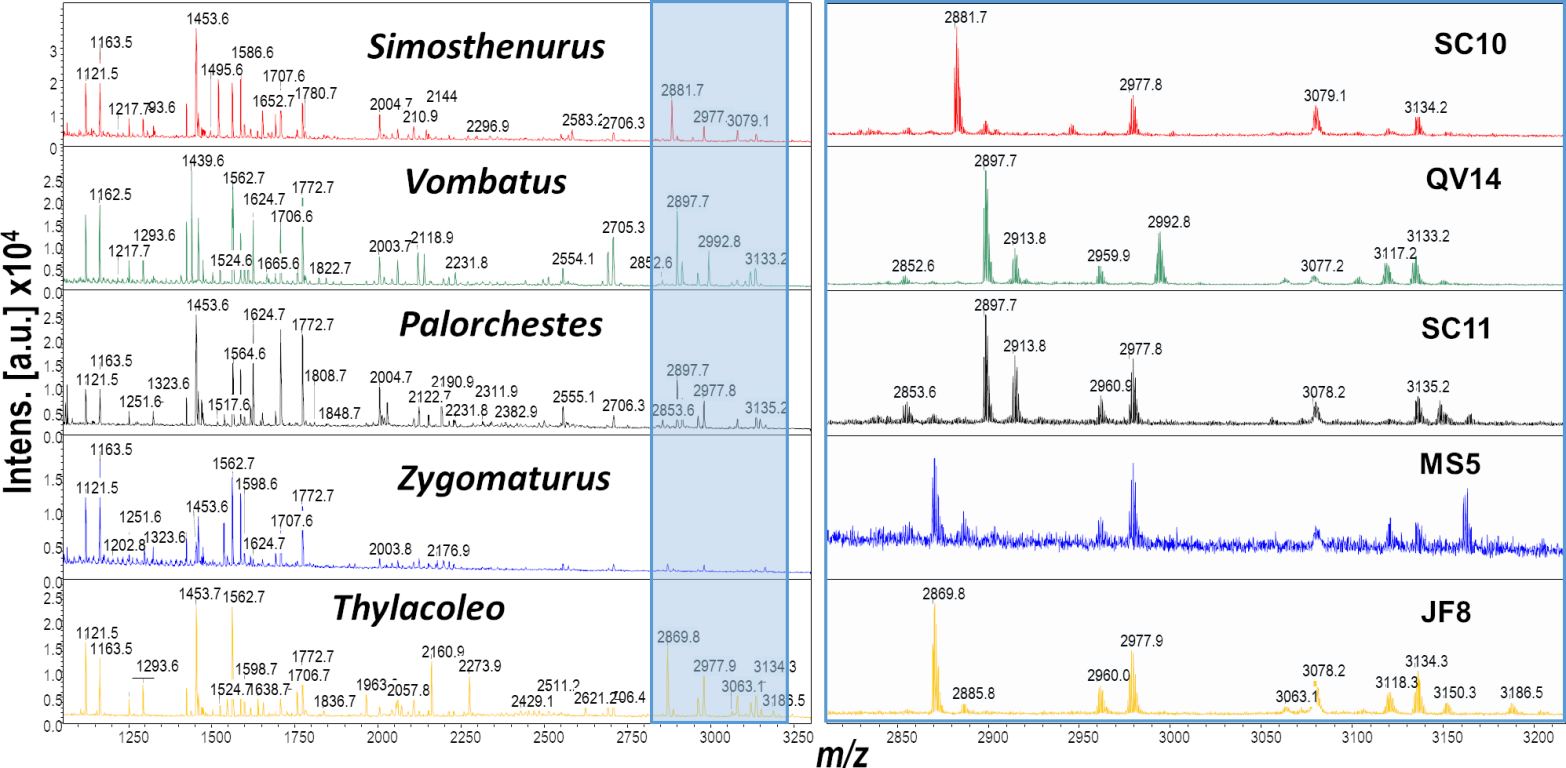
Total collagen peptide mass fingerprints (left) and zoomed-in component (right, showing expanded detail of section shaded blue on the left image) to enhance the higher mass/charge (*m/z*) range of peptides in the *Zygomaturus* spectrum (*Simosthenurus* comparison to four other macropodids shown in electronic supplementary material, figure S5; tandem MS spectra shown as electronic supplementary material, figures S8–S13).

**Figure 2.**
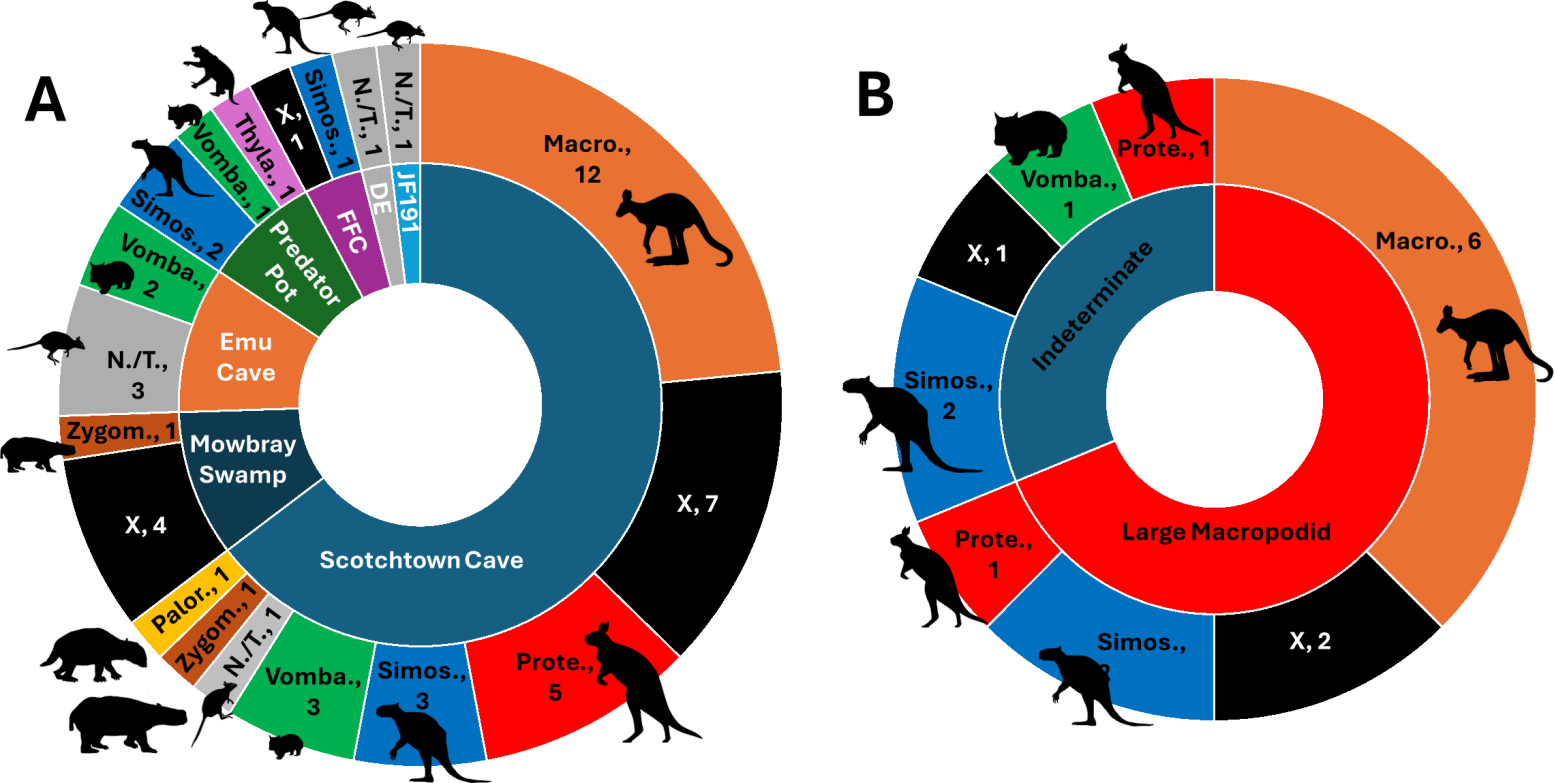
Species composition based on ZooMS interpretation (electronic supplementary material, table S2) for (A) all samples per site and (B) fragmented samples from Scotchtown Cave which could not be identified morphologically to species or genus. Macro. = *Macropus*, N./T. = *Notamacropus*/*Thylogale*, Palor. = *Palorchestes*, Prote. = *Protemnodon*, Simos. = *Simosthenurus*, Vomba. = *Vombatus*, Zygom. = *Zygomaturus*, X = Poor, FFC = Femur Fest Cave, DE = Devil’s Earhole. Silhouettes taken from https://www.phylopic.org.

**Figure 3.**
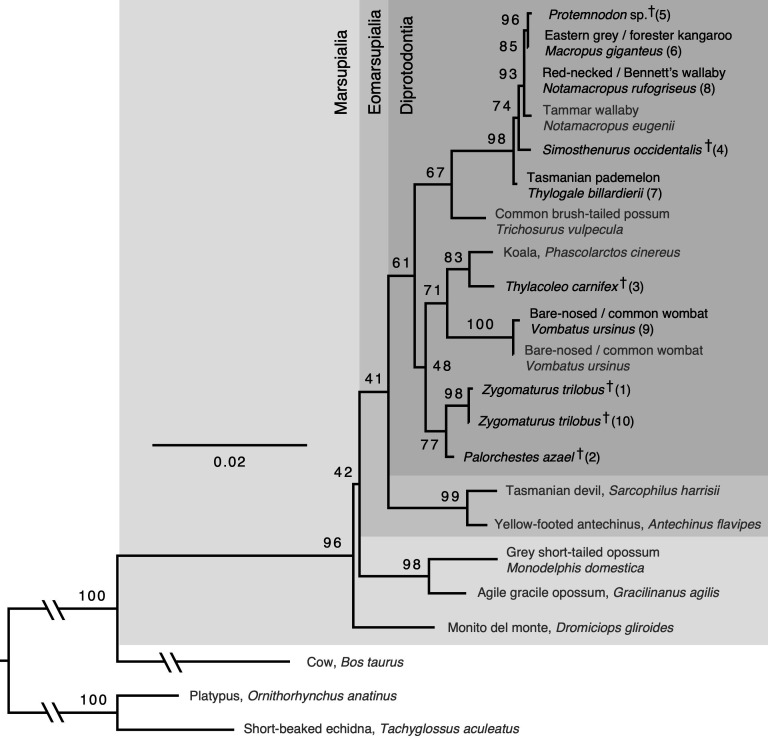
Maximum likelihood consensus tree based on our full amino acid alignment, built using IQ-TREE. Branch lengths are proportional to number of amino acid substitutions. Scale is in substitutions per site. Numbers associated with branches represent ultrafast bootstrap support (%). Species represented by amino acid sequences extracted from published genomes are labelled in grey; species represented by new amino acid sequences generated in this study are labelled in black (and numbered according to the order in which they were processed by LC-MS/MS). Extinct species are marked with †.

The preservation state of collagen, as assessed via PMF, varied across the 51 samples in this study (electronic supplementary material, table S2). However, at least one specimen per taxon that had a confident morphological identification yielded good enough collagen (i.e. high *m/z* peaks visible) to warrant further in-depth sequencing via LC-MS/MS (figure 1; electronic supplementary material, figure S8). The success rates were consistent with geological locality/site type. For example, only one in five of the specimens from Mowbray Swamp (and even this one, MS5, had noticeably poorer collagen than from specimens of others sites; see figure 1) whereas 19 of the 33 specimens (i.e. >50%) were successful for Scotchtown Cave, and all the material from Emu Cave, Predator Pot, Devils Earhole and JF191 yielded positive results (figure 2). There was no apparent relationship between collagen decay (measured *m/z* 1105 : 1106 ranging from 0.58−0.77) and the archaeological sites, with the relatively poorly preserved remains of the Mowbray Swamp yielding one of the lowest measures (0.62) of deamidation despite yielding relatively low intensity high *m/z* peaks (figure 3; electronic supplementary material, table S2).

Due to the highly conserved nature of the marsupial bone collagen sequences, there are a large number of shared peaks (i.e. peptides [23,24]); the vast majority of peaks were readily identified through LC-MS/MS matches to sequences of extant species using standard search conditions (i.e. not seeking novel sequences through error tolerant searches; electronic supplementary material, tables S8–S17). For example, the *m/z* 2869 peak of the extinct *Zygomaturus* and *Thylacoleo* not seen elsewhere in taxa of this study was confidently matched to the peptide sequence (GLTGPIGPPGPAGPSGDKGESGPSGPAGPTGAR; amino acids COL1ɑ1 603−635) present in DNA-derived sequences of both *Phascolarctos* and *Sarcophilus* (there appear to be at least 18 substitutions between these taxa as evidenced by the LC-MS/MS data; electronic supplementary material, table S18). Nonetheless, with the exception of *Simosthenurus* (molecular markers for a collagen fingerprint identification confidently established elsewhere [23]), all the specimens subject to LC-MS/MS analyses were of morphologically identified specimens.

### (c) Collagen (I) molecular phylogeny

**Figure 4.**
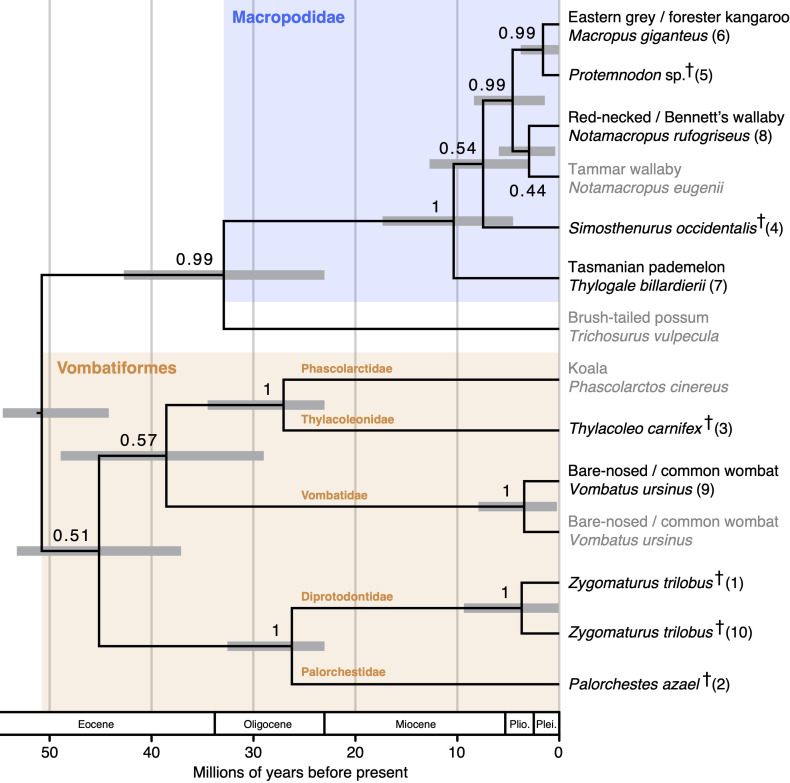
Bayesian maximum clade credibility tree based on diprotodontian amino acid sequences, built using BEAST2. Branch lengths are proportional to time (millions of years before present). Node heights represent mean age estimates; bars associated with nodes represent 95% highest posterior densities for node age estimates. Numbers associated with branches represent posterior probabilities. Species represented by amino acid sequences extracted from published genomes are labelled in grey; species represented by new amino acid sequences generated in this study are labelled in black (and numbered according to the order in which they were processed). Extinct species are marked with †.

Phylogenetic relationships between extant species inferred using our amino acid dataset largely agree with published results obtained using nucleotide data [65,66], though generally our results recovered these relationships with lower confidence (figures 3 and 4). The major exceptions to this low confidence were the monophyly of Marsupialia and Macropodidae, which received strong support (96% and 98% bootstrap support, respectively). In contrast, other recognized clades such as Eomarsupialia (i.e. the Australian marsupials) and Diprotodontia were monophyletic in the best maximum likelihood tree, but with only marginal support (41% and 61% bootstrap support, respectively). This lower confidence compared to studies based on nucleotide data is likely to be due to a relatively low number of variable sites in our alignment (i.e. several hundred) compared to conventional nucleotide datasets (which may include from thousands up to millions of variable sites), as well as selective constraints on collagen peptides limiting the breadth of possible substitutions.

Our *Simosthenurus* and *Protemnodon* amino acid sequences (76% and 83% sequence coverage, respectively) both formed part of a strongly supported Macropodidae (98% bootstrap support; 1.0 posterior probability). Within Macropodidae, *Protemnodon* was the sister-taxon to *Macropus* (96% bootstrap support; 0.99 posterior probability); however, the position of *Simosthenurus* was poorly resolved, forming a clade with *Macropus* (79% coverage), *Notamacropus* (84% coverage) and *Protemnodon* (74% bootstrap support; 0.54 posterior probability) to the exclusion of *Thylogale*. Our sequences for *Thylacoleo* (82% coverage), *Zygomaturus* (75% coverage) and *Palorchestes* (78% coverage) all formed a clade with the extant vombatiforms (*Vombatus*, *Phascolarctos*), though monophyly of this clade was only weakly supported (48% bootstrap support; 0.51 posterior probability). Within Vombatiformes, *Thylacoleo* was moderately well supported as the sister-taxon to *Phascolarctos* (83% bootstrap support; 1.0 posterior probability), whereas *Zygomaturus* and *Palorchestes* formed a moderately well-supported clade (77% bootstrap support; 1.0 posterior probability) that was sister-taxon to a clade comprising the remaining vombatiforms (71% bootstrap support; 0.57 posterior probability).

Using Shimodaira’s [51] approximately unbiased test, we were able to reject several alternative phylogenetic positions for *Thylacoleo*, *Palorchestes* and *Zygomaturus*. For example, we were able to reject (at α = 0.05) a sister-taxon relationship between *Thylacoleo* and *Vombatus* (*p* = 0.009), *Thylacoleo* and all other diprotodontians (*p* = 0.031), *Thylacoleo* and a clade comprising the non-vombatiform diprotodontians (*p* = 0.022) and *Thylacoleo* and a clade comprising all other vombatiforms (*p* = 0.012). However, we could not reject alternatives where *Thylacoleo* was sister to a clade comprising *Phascolarctos* and *Vombatus* (*p* = 0.401), or a clade comprising *Palorchestes* and *Zygomaturus* (*p* = 0.062), though the latter of these alternatives had a substantially lower likelihood compared to the best tree (Δ*L* = 19.30; electronic supplementary material, table S4). Several alternative positions for *Palorchestes* and *Zygomaturus* could also not be rejected, including some with only marginally lower likelihoods compared to the best tree (e.g. a clade comprising *Palorchestes* and *Zygomaturus* as sister to all other diprotodontians; Δ*L* = 2.61, *p* = 0.607; electronic supplementary material, table S4).

Age estimates (figure 4) for the common ancestors of *Thylacoleo* and *Phascolarctos* (95% highest posterior density, HPD = 23.03–34.5 Ma), *Zygomaturus* and *Palorchestes* (95% HPD = 23.03–32.56), Macropodidae and *Trichosurus* (95% HPD = 23.04–42.72) were relatively imprecise and abutted the minimum bound placed on these nodes (i.e. 23.03 Ma); the majority of the posterior density for the age of these nodes overlapped the Oligocene. In contrast, most of the posterior density for the age of the crown of Vombatiformes (95% HPD = 37.1–53.22 Ma) and the clade comprising *Phascolarctos*, *Thylacoleo* and *Vombatus* (95% HPD = 28.99 = 48.92 Ma) overlapped the Eocene, whereas the crown age of Macropodidae (95% HPD = 4.54–17.34 Ma) overlapped the Middle to Late Miocene.

## 4. Discussion

### (a) Collagen as a biomolecular resource for reaching deeper into the past

The successful recovery of a collagen fingerprint from a ~100 ka short-faced kangaroo (*S. occidentalis*) dentary from Femur Fest Cave demonstrates that PMF can be used to confidently identify faunal remains dating to the critical period of change coinciding with human arrival and megafauna extinction in Sahul (i.e. approx. 50–100 ka). This suggests that ZooMS can be used to expand the number of confidently identified megafauna remains available for use in the construction of extinction chronologies, permitting more precise estimates for extinction ages of individual taxa and the length of human-megafauna coexistence in Sahul—key variables for testing extinction hypotheses [4]. Importantly, the ability to identify highly fragmented bone material—possibly including human-processed remains—could provide an additional line of evidence to investigate early interactions (or lack thereof) between people and megafauna in Sahul. This is demonstrated by our finding that four of five bone fragments of morphologically indeterminate species, and 10 of 13 speculative morphological identifications, could be unambiguously assigned to a taxon using ZooMS (figure 2B; electronic supplementary material, table S2; noting exception for *Notamacropus/Thylogale* that could not be distinguished). However, of the 51 samples in our analysis, we were unable to resolve the taxonomic identity of 12 samples (figure 2; electronic supplementary material, table S2), due to poor collagen preservation in all cases.

Bones from three other Pleistocene age caves (Scotchtown Cave, Predator Pot, Emu Cave) yielded collagen suitable for ZooMS in 26 of 42 samples. Although the age of these fossils is not tightly constrained, they contain extinct megafauna and are unlikely to be much younger than ~40 ka. This finding reinforces the evidence that karst caves in cool temperate environments are favourable for the preservation of collagen within the timeframe pertinent to megafauna extinction in Sahul. Bones from Mowbray Swamp returned a lower proportion of positive results with only one sample successfully fingerprinted. These bones may be older than most other cave material analysed in this study by tens of thousands of years and the lower return may reflect their greater age. This factor is probably compounded by taphonomic effects related to prolonged immersion in acidic waterlogged peat, which have also complicated attempts to directly date bones from Mowbray Swamp [67]. However, the small number of samples in our analysis (*n* = 5) and the fact that *Z. trilobus* collagen was recovered in one case suggests that swamp fossils should not be dismissed as candidate material for retrieving molecular information, whether this is ZooMS fingerprinting for species identification, or more in-depth analyses of peptide sequencing for phylogenetic inferences.

### (b) ZooMS applications in megafaunal extinction debates

Although there are cases throughout the world where the causes of megafaunal extinction are less ambiguous, including cases where humans are clearly implicated, the debate about the causes of megafaunal extinction in Sahul continues [17,68], primarily due to disagreements over the strength of available evidence for extinction timing. For example, an argument has been made implicating humans in the extinction of megafauna from Sahul based on direct radiocarbon ages and OSL ages from a small sample of non-archaeological bone and sediment from Tasmanian caves, which demonstrated temporal overlap between people and megafauna [39]. However, Cosgrove *et al*. [69] questioned this conclusion by pointing out that, with one exception, Turney *et al*.’s [39] data did not confirm actual temporal overlap between megafauna species and humans in Tasmania. The rebuttal depends on an ‘absence of evidence’ argument, which highlights the difficulty shared by both sides in this debate of extracting robust conclusions from the scanty evidence presently available. Probabilistic modelling of the same data [70] does not address the fundamental constraint imposed by the paucity of data. Scope exists for a handful of confidently identified and dated megafauna fossils to tip the balance of evidence in favour of either side in this debate.

Historically, the dating of last occurrences of megafauna in Tasmania, and Sahul more broadly, has depended on the availability of fossils that satisfy the two basic criteria of being identifiably megafauna and being dateable. For the latter, there is a strong preference for bones containing sufficient collagen to be directly dated using the radiocarbon method, as opposed to dated contextually using associated materials with attendant potential for intrusion of younger or older materials. This requirement has excluded fragmented or otherwise degraded bones excavated from archaeological and palaeontological contexts. For example, 90% of 256 200 bones considered in a faunal analysis of the Late Pleistocene human occupation site of Kutikina Cave, Tasmania, could not be identified to either taxon or body part [71]. Similarly, between 33% and 73% of bones from five other Late Pleistocene human occupation sites in Tasmania have been resolved only to ‘large mammal’ or ‘large macropod’ level [72]. This raises the possibility that reliance on morphologically intact remains could bias the available evidence towards environmental change as the primary cause of extinction, rather than human-driven causes. Therefore, testing these hypotheses requires a new type of evidence, and ZooMS collagen PMF offers a solution by enabling the identification of fragmentary faunal remains through increasing knowledge on collectively unique markers (e.g. electronic supplementary material, table S19).

### (c) Sequencing ancient collagen for inferring the relationships between extinct marsupial megafauna

The topologies of the consensus trees obtained from our maximum-likelihood and Bayesian analyses (figures 3 and 4) are highly concordant with comparable phylogenies obtained using nucleotide data [65,66], including strong support for a clade comprising *Protemnodon*, *Simosthenurus* and the extant macropodids (Macropodidae), within which *Protemnodon* is closely allied to extant *Macropus* and *Notamacropus* kangaroos [8,31]. However, our data are equivocal on the widely accepted split between Sthenurinae (represented by *Simosthenurus*) and Macropodinae (represented by *Thylogale*, *Macropus*, *Notamacropus* and *Protemnodon*). In addition, our age estimate for the origin of the *Simosthenurus* lineage (3.0−12.7 Ma), while compatible with the earliest fossil evidence for both sthenurines and macropodines in the late Miocene (5.2−10.4 Ma [27]), is substantially younger than estimates based on nucleotide data (e.g. 16.6−22.6 Ma [31]). Indeed, Buckley *et al*. [36] similarly obtained underestimates (compared to nucleotide data) for several nodes within the eutherian mammal phylogeny, perhaps suggesting that collagen peptide evolution may not always be strictly clocklike. In contrast, our age estimate for the common ancestor of *Thylogale*, *Macropus*, *Notamacropus* and *Protemnodon* (4.5−17.3 Ma) is broadly consistent with estimates based on nucleotide data (9.3−13.2 Ma [31]; 6.5−8.4 Ma [66]), though with greater uncertainty (probably due at least partly to a relatively low number of variable amino acids). Collectively, these results suggest that our data—while providing lower topological resolution, and decreased temporal accuracy and precision compared to nucleotide data—still encompass sufficient phylogenetic signal to provide meaningful (though not conclusive) insights into the evolutionary history of *Thylacoleo*, *Zygomaturus* and *Palorchestes*.

Our results suggest a novel hypothesis for the phylogenetic position of *Thylacoleo*, as a closer relative to the koala (*Phascolarctos cinereus*; the only living member of Phascolarctidae) than to any other taxon in our dataset. While most recent authors agree that Thylacoleonidae is part of Vombatiformes, our result is notable because previous studies have usually positioned Thylacoleonidae either as the sister-taxon to a clade comprising all other vombatiform lineages [28,29] or as part of a clade with other vombatiforms to the exclusion of Phascolarctidae (i.e. ‘Vombatimorphia’) [27,73,74], but apparently never as sister-taxon to Phascolarctidae. Equally unexpected is our relatively young age estimate for the common ancestor of Thylacoleonidae and Phascolarctidae: 23.0−34.5 Ma. This timeline would suggest that the distinctive traits of these families—including adaptations for carnivory in thylacoleonids—evolved relatively soon before the first appearance of fossils belonging to these lineages in the late Oligocene. Alternatively, however, this age may be an underestimate, perhaps as a result of ‘non-clock-like’ collagen peptide evolution. In any case, in Beck *et al*.’s [29] character matrix, strong crest-like enamel crenulations (their character 15) are noted as being present in most fossil thylacoleonids (including *Thylacoleo carnifex*), most fossil phascolarctids and the modern koala. Where data were available, all other vombatiforms and outgroups were noted as having weak or absent enamel crenulations [29], perhaps indicating that strong crest-like enamel crenulations evolved in (or were retained by) the common ancestor of thylacoleonids and phascolarctids after its divergence from other diprotodontian lineages. Though alternative phylogenetic hypotheses cannot be rejected based on these peptide data alone, our evidence for a sister-taxon relationship between Thylacoleonidae and Phascolarctidae provides a new perspective on the phylogeny and morphological trait evolution of fossil vombatiforms that warrants more comprehensive investigation.

We also show that the bone collagen phylogenetic signal is consistent with a sister-taxon relationship between Diprotodontidae (*Zygomaturus*) and Palorchestidae (*Palorchestes*), in agreement with most phylogenies based on morphological traits [27,29]. Again, our age estimate for the common ancestor of Diprotodontidae and Palorchestidae is relatively young (23.0−32.6 Ma) suggesting—if correct—that the distinctive traits of these families evolved soon before their first appearances in the fossil record. Indeed, the unique rostral morphology of *Palorchestes* was already evident in the earliest known palorchestid crania [75,76]. Support for the position of the clade comprising *Zygomaturus* and *Palorchestes* is low, possibly due to a relatively high number of unresolved residues in their respective sequences (22–26%; whereas sequences for some other species had <20%), or simply short internodes coupled with a relatively low total number of variable amino acids. Regardless, collagen peptide sequences are unlikely to assist with establishing the relationships between Diprotodontidae, Palorchestidae and the other fossil vombatiform families—Ilariidae, Wynyardiidae, Maradidae and Mukupirnidae—because the latter taxa became extinct by the early Miocene at the latest [27], well beyond the temporal range of readily retrievable preserved bone protein [77,78]. However, collagen peptide sequences may be useful for exploring the relationships between other extinct Pleistocene vombatiform genera (e.g. *Diprotodon*), including those from New Guinea (e.g. *Maokopia*, *Hulitherium*).

## 5. Conclusions

Although the use of collagen fingerprinting for taxonomic identification is now well established, this is the first instance in which it has been shown to be successful in marsupial megafauna of such antiquity (i.e. approx. 100 ka). By allowing identification of megafauna remains that pre-date the arrival of humans in Sahul, ZooMS offers great potential utility in the ongoing human versus climate-caused megafaunal extinction debate. Importantly, the amenability of ZooMS to high-throughput methodology enables quick processing (and identification) of many thousands of specimens [21,79,80]. In doing so, it could also be used to screen and evaluate extensive numbers of archaeological and palaeontological specimens for radiocarbon dating potential [34], improving chronologies further still. In addition, the identification of a much greater number of specimens from sites dated through other means, such as OSL, would also be valuable for progressing the debate about megafaunal extinction. The use of ZooMS will ultimately lead to improving our understanding of faunal compositions at archaeological sites associated with human activity and improving the resolution of records of biodiversity change.

Beyond ZooMS, our in-depth analysis of collagen peptide sequences through LC-MS/MS sequencing allowed us to undertake molecular phylogenetic analyses that, for the first time, included biomolecular sequence information from the enigmatic extinct marsupial megafauna *Thylacoleo, Zygomaturus* and *Palorchestes.* Though other hypotheses could not be rejected, the weight of evidence provided by the collagen peptide data favoured a close relationship between *Thylacoleo* (the so-called ‘marsupial lion’) and the koala, as part of a broader clade including wombats and the extinct genera *Zygomaturus* and *Palorchestes*. The increasing use of collagen to retrieve phylogenetic information—as one among many tools—offers great potential to better understand the diversity and evolution of past life on Earth.

## Data Availability

All proteomic data, both peptide mass fingerprints (as .mzXML files) and LC-MS/MS sequencing data (as .MGF files) are made available in Figshare at [81] along with alignment files for the phylogenetic analyses. The remaining data, as well as presentations of important aspects of the results presented in the electronic supplementary material, text, figures and tables. Supplementary material is available online [82].
